# Energy intake during hospital stay predicts all-cause mortality after discharge independently of nutritional status in elderly heart failure patients

**DOI:** 10.1007/s00392-020-01774-y

**Published:** 2021-01-05

**Authors:** Satoshi Katano, Toshiyuki Yano, Hidemichi Kouzu, Katsuhiko Ohori, Kanako Shimomura, Suguru Honma, Ryohei Nagaoka, Takuya Inoue, Yuhei Takamura, Tomoyuki Ishigo, Ayako Watanabe, Masayuki Koyama, Nobutaka Nagano, Takefumi Fujito, Ryo Nishikawa, Wataru Ohwada, Akiyoshi Hashimoto, Masaki Katayose, Tetsuji Miura

**Affiliations:** 1 Division of Rehabilitation, Sapporo Medical University Hospital, Sapporo, Japan; 2 Department of Cardiovascular, Renal and Metabolic Medicine, Sapporo Medical University School of Medicine, Sapporo, Japan; 3 Department of Cardiology, Hokkaido Cardiovascular Hospital, Sapporo, Japan; 4 Department of Rehabilitation, Hakodate Goryokaku Hospital, Hakodate, Japan; 5 Department of Rehabilitation, Sapporo Cardiovascular Hospital, Sapporo, Japan; 6 Division of Rehabilitation, Hokuto Hospital, Obihiro, Japan; 7 Department of Rehabilitation, Hokkaido Ohno Memorial Hospital, Sapporo, Japan; 8 Division of Hospital Pharmacy, Sapporo Medical University Hospital, Sapporo, Japan; 9 Division of Nursing, Sapporo Medical University Hospital, Sapporo, Japan; 10 Department of Public Health, Sapporo Medical University School of Medicine, Sapporo, Japan; 11 Division of Health Care Administration and Management, Sapporo Medical University School of Medicine, Sapporo, Japan; 12 Second Division of Physical Therapy, School of Health Sciences, Sapporo Medical University, Sapporo, Japan

**Keywords:** Heart failure, Nutrition, MNA-SF, Energy intake, Mortality, Elderly

## Abstract

**Objective:**

Malnutrition is associated with an increased risk of mortality in heart failure (HF) patients. Here, we examined the hypothesis that assessment of energy intake in addition to nutritional status improves the stratification of mortality risk in elderly HF patients.

**Methods:**

We retrospectively examined 419 HF patients aged ≥ 65 years (median 78 years, 49% female). Nutritional status was assessed by the Mini Nutritional Assessment Short Form (MNA-SF), and daily energy intake was calculated from intake during 3 consecutive days before discharge.

**Results:**

During a median 1.52-year period (IQR 0.96–2.94 years), 110 patients (26%) died. Kaplan–Meier survival curves showed that patients with low tertile of daily energy intake had a higher mortality rate than did patients with high or middle tertile of daily energy intake. In multivariate Cox regression analyses, low daily energy intake was independently associated with higher mortality after adjustment for the model including age, sex, BNP, Charlson Comorbidity Index, history of HF hospitalization, and cachexia in addition to MNA-SF. Inclusion of both MNA-SF and energy intake into the adjustment model improved the accuracy of prediction of the mortality after discharge (continuous net reclassification improvement, 0.355, *p* = 0.003; integrated discrimination improvement, 0.029, *p* = 0.003). Results of a fully adjusted dose-dependent association analysis showed that risk of all-cause mortality was lowest among HF patients who consumed 31.5 kcal/kg/day of energy.

**Conclusions:**

Energy intake during hospital stay is an independent predictor of the mortality in elderly HF patients, and its assessment together with established predictors improves the mortality risk stratification.

**Graphic abstract:**

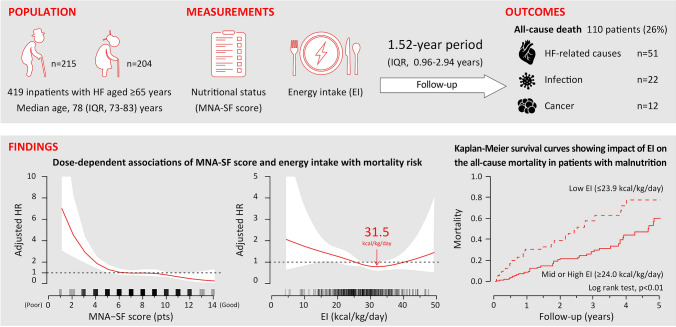

## Introduction

Heart failure (HF) is a major public health problem that affects 1–2% of adults, and it is a leading cause of morbidity and mortality [1, 2]. In addition to cardiac dysfunction per se, the presence of comorbidities such as chronic kidney disease (CKD), anemia, and cachexia is associated with poor prognosis [3, 4]. Among them, malnutrition has received much attention since it is frequently observed in HF patients, especially in elderly HF patients [5–7]. In addition, results of several studies have shown that malnutrition is an independent predictor of the all-cause death and cardiac events in HF patients [5–7]. Several tools for screening and/or assessment of nutrition have been developed: biochemistry-based screening tools such as prognostic nutritional index and multidimensional nutritional screening tools such as the Mini Nutritional Assessment (MNA) and the MNA-Short Form (MNA-SF). Currently, MNA or MNA-SF is recommended as an appropriate nutritional screening test for elderly people by the European Society for Clinician Nutrition and Metabolism (ESPEN) [8, 9]. Furthermore, the results of a recent meta-analysis suggest that MNA or MNA-SF is superior to biochemistry-based screening tools for prediction of the mortality in HF patients [5].

The cause of malnutrition in HF is thought to be multifactorial. Insufficient energy intake for energy requirement, malabsorption, and increased catabolic state play pivotal roles in HF-induced malnutrition, and the causal factors are aggravated by comorbidities of HF such as CKD and diabetes mellitus and by adverse reactions of pharmacological agents [10–12]. Importantly, the factors leading to HF-induced malnutrition are at least partly modifiable by optimal HF therapy. However, reversibility of the nutritional status is not considered in the assessment of nutritional status during the period of hospitalization. Low energy and protein intake are undoubtedly associated with increased risk of malnutrition. Indeed, the results of previous studies demonstrated that an appropriate amount of energy intake including its intentional achievement during hospital stay is associated with better survival after discharge in critically ill patients, especially in patients who are malnourished [13]. However, it has not been clear whether the nutrition state and energy intake are independent predictors of the mortality in HF patients.

The aim of this study was to demonstrate the impact of energy intake on prediction of all-cause death in elderly HF patients. In addition, we analyzed the dose-dependent association between daily energy intake and mortality since there is no guideline showing optimal energy intake for lowering mortality in HF patients.

## Methods

### Study subjects

**Fig. 1:**
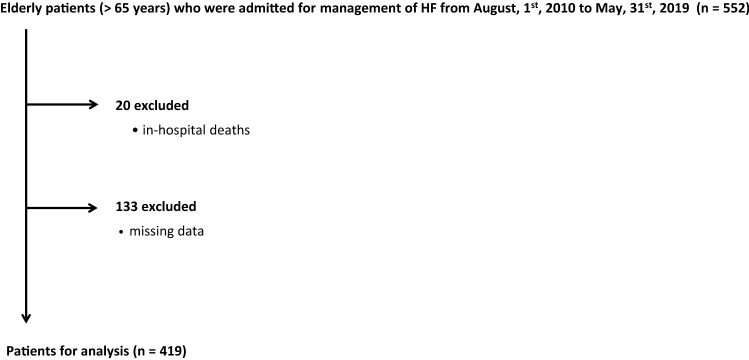
Flow chart of the inclusion of the study subjects

This study was a single-center, retrospective, and observational study. We retrospectively enrolled consecutive patients aged ≥ 65 years who were admitted to our institute for management of HF during the period from August 1, 2010 to May 31, 2019 (Fig. 1). This period was selected for the enrollment of the study subjects since routine assessment of both nutritional status and energy intake was commenced on August 1, 2010. HF was diagnosed by cardiologists according to the Framingham criteria [14]. Exclusion criteria were death during hospitalization and data missing during the follow-up period. This study was conducted in strict adherence with the principles of the Declaration of Helsinki and was approved by the Clinical Investigation Ethics Committee of Sapporo Medical University Hospital (number 302-243).

### Assessment of nutritional status and energy intake

Nutritional status was assessed by MNA-SF, and daily energy intake was calculated from intake during 3 consecutive days before discharge.

Nutritional status was assessed using the MNA-SF as previously described [9, 15]. The MNA-SF consists of 6 questions about reduction in food intake over the past 3 months, weight loss during the past 3 months, mobility, psychological stress or acute disease in the past 3 months, neuropsychological problems, and BMI and it is scored 0–14. Nutritional status is categorized to normal nutritional status, at risk of malnutrition, and malnourished by the MNA-SF score of 12–14, 8–11, and 0–7, respectively.

Daily energy intake was estimated as previously reported [15]. The patients took 1400–2200 kcal meals depending on their standard body weight during hospital stay. Physical therapists and nursing staff recorded a visual estimate of the percentage of each item that patients ate during 3 consecutive days before discharge and then calculated the amount of energy intake per day. The visual estimation of energy intake by nursing staff has been shown to have a good correlation with the estimation by weighing dietary intake [16]. Daily energy intake (kcal/kg/day) can be normalized by actual body weight, standard body weight, or target body weight at the time of discharge. In this study, we primarily used daily energy intake per actual body weight. In addition, relationships between daily energy intake with nutritional status and other parameters were examined also by use of daily energy intake per standard body weight and that per target body weight since normalization of energy intake by actual body weight may cause spurious estimation of the energy intake in overweight or underweight patients [17, 18]. Standard body weight was calculated as follows: 22 kg/m^2^ × (height [m])^2^. Target body weights for Japanese people were set to 23.5 kg/m^2^ × (height [m])^2^ for patients aged < 75 years and to 25.0 kg/m^2^ × (height [m])^2^ for patients aged ≥ 75 years as previously reported [19].

### Laboratory data and echocardiography

Data for brain natriuretic peptide (BNP), serum albumin, hemoglobin, uric acid, creatinine, creatinine-based estimated glomerular filtration rate (eGFRcre), sodium, and total lymphocyte count were obtained within 7 days of assessment of nutritional status. Transthoracic echocardiography was performed by the standard protocol, and the left ventricular ejection fraction (LVEF) was measured by the modified Simpson method. Heart failure with reduced ejection fraction (HFrEF) was defined as LVEF of less than 40%.

### Comorbidities

The existence of comorbidities was assessed on the basis of medical information including the patient’s history, data, and prescribed drugs. Comorbidities were assessed by the use of the Charlson Comorbidity Index [20], taking into account the following 19 comorbid conditions: myocardial infarction, congestive HF, peripheral artery disease, cerebrovascular disease, dementia, chronic pulmonary disease, connective tissue diseases, rheumatic disease, peptic ulcer disease, mild liver disease, diabetes mellitus with or without chronic complication, hemiplegia/paraplegia, renal disease, any malignancy without metastasis, leukemia, lymphoma, moderate or severe liver disease, metastatic solid tumor, and HIV infection. According to the criteria by Fearon et al., cachexia was diagnosed when HF patients had any of following factors: more than 5% loss of stable body weight over the past 6 months, a BMI less than 20 kg/m^2^ and ongoing weight loss of more than 2%, or sarcopenia and ongoing weight loss of more than 2% [21]. Chronic kidney disease (CKD) was defined as eGFRcre being less than 60 mL/min/1.73 m^2^.

### Clinical endpoint

The clinical endpoint was all-cause death during the follow-up period from the day of discharge until February 29, 2020. Data for the clinical endpoint in the enrolled patients were collected from medical records.

### Statistical analysis

Data are presented as means ± standard deviation or medians (interquartile range [IQR] 25th–75th percentiles) depending on the results of the Shapiro-Wilk test. Baseline characteristics were compared by one-way analysis of variance, the Kruskal–Wallis test, or the Chi-square test where appropriate.

Survival curves were calculated by the Kaplan–Meier method, and statistical significance of differences between the curves was assessed by the log-rank statistics. Univariate and multivariate Cox proportional hazards analyses were used to evaluate prognostic predictive ability. The dose-dependent associations of MNA-SF score and total energy intake with mortality risk were examined using a Cox regression model with a restricted cubic spline function with four knots.

According to adjustment of variables in the Cox regression models, we constructed logistic models for all-cause death. Harrell’s C-index was calculated and compared between the base model and the model with the addition of MNA-SF score and daily energy intake according to the methods of DeLong et al. [22]. Furthermore, to examine the significance of the incremental discriminative value added by MNA-SF score and daily energy intake, we calculated the log-likelihood ratio (LLR), continuous net reclassification improvement (cNRI) and integrated discrimination improvement (IDI) [23].

A two tailed *p* value < 0.05 was considered statistically significant. Statistical analyses were performed using JMP version 14.3.0 (SAS Institute Inc., Cary, NC, USA) and R version 3.6.2 (R Foundation for Statistical Computing, Vienna, Austria).

## Results

Of 552 HF patients initially screened, 153 patients were excluded by the exclusion criteria, and data for 419 patients were used for analyses as shown in Fig. 1.

### Baseline characteristics

As shown in Table 1, the median age of the patients was 78 years (IQR 72–83 years) and 49% of them were female. The median BMI of the patients was 21.1 kg/m^2^. At the time of discharge, 59, 31, and 5% of the patients were in NYHA functional class II, III, and IV, respectively. The median LVEF was 48% (IQR 34–63%), and 34% of the patients had HFrEF. Fifty-one percent of the patients had a prior history of hospitalization for HF. The most frequent etiology of HF was valvular heart disease (33%) followed by cardiomyopathy (27%) and ischemic heart disease (21%). The median Charlson Comorbidity Index of the patients was 5 points (IQR 4–7 points).

**Table 1:** Baseline characteristics according to the standard category of nutritional status by MNA-SF scores

Variables	All		MNA-SF score						
			Malnutrition		At risk		Normal		*p* value
			≤ 7 points		8–11 points		≥ 12 points		
	*N* = 419		*N* = 217		*N* = 168		*N* = 34		
Age, years	78	(72, 83)	77	(72, 83)	78	(72, 83)	78	(71, 82)	0.848
≥ 75 years, *N* (%)	257	(61)	132	(61)	104	(62)	21	(62)	0.976
Female, *N* (%)	204	(49)	105	(48)	92	(55)	18	(53)	0.454
Height, cm	157	± 9	157	± 9	157	± 8	156	± 10	0.827
Body weight, kg	51.6	(44.5, 59.8)	47.8	(41.8, 53.7)	55.5	(49.2, 64.6)	58.7	(53.1, 65.8)	<0.001
BMI, kg/m^*2*^	21.1	(18.6, 23.4)	19.3	(17.5, 21.7)	22.7	(20.5, 24.9)	23.6	(21.6, 26.7)	<0.001
Heart rate, bpm	68	(60, 75)	69	(61, 77)	66	(60, 74)	65	(56, 70)	0.004
Systolic blood pressure, mmHg	116	(103, 129)	113	(101, 126)	118	(106, 131)	121	(112, 134)	0.004
NYHA functional class, *N* (%)									0.009
I	23	(6)	6	(3)	12	(7)	5	(15)	
II	245	(59)	121	(56)	104	(62)	20	(59)	
III	129	(31)	73	(34)	47	(28)	9	(26)	
IV	22	(5)	17	(8)	5	(3)	0	(0)	
LVEF, %	47.6	(34.1, 62.7)	45.7	(31.3, 60.9)	50.2	(37.5, 63.4)	58.4	(33.3, 63.9)	0.094
< 40%,* N* (%)	142	(34)	86	(40)	47	(28)	9	(26)	0.036
Smoking history,* N* (%)	130	(31)	64	(29)	50	(30)	16	(47)	0.108
Barthel Index score, points	85	(75, 90)	80	(70, 90)	85	(80, 95)	90	(85, 95)	<0.001
History of HF hospitalization,* N* (%)	207	(51)	119	(55)	76	(45)	12	(35)	0.040
Etiology,* N* (%)									0.411
Valvular heart disease	140	(33)	71	(33)	55	(33)	14	(41)	
Cardiomyopathy	114	(27)	53	(24)	50	(30)	11	(32)	
Ischemic	87	(21)	53	(24)	29	(17)	5	(15)	
Device,* N* (%)									0.539
Pacemaker	62	(15)	37	(17)	22	(13)	3	(9)	
ICD	31	(8)	15	(7)	14	(8)	2	(6)	
CRT-P or CRT-D	28	(7)	18	(8)	8	(5)	2	(6)	
Comorbidity,* N* (%)									
Hypertension	299	(71)	148	(68)	126	(75)	25	(74)	0.329
Diabetes mellitus	190	(45)	105	(48)	75	(45)	10	(29)	0.115
Dyslipidemia	226	(54)	114	(53)	90	(54)	22	(65)	0.413
Chronic kidney disease	312	(75)	166	(77)	128	(76)	18	(53)	0.011
Atrial fibrillation	189	(45)	98	(45)	76	(45)	15	(44)	0.993
Chronic pulmonary disease	100	(24)	63	(29)	30	(18)	7	(21)	0.035
History of cancer	108	(26)	57	(26)	43	(26)	8	(24)	0.942
Orthopedic disorder	132	(32)	69	(32)	49	(29)	14	(41)	0.385
Cachexia	41	(10)	34	(16)	7	(4)	0	(0)	<0.001
Charlson Comorbidity Index, points	5	(4, 7)	5	(4, 7)	5	(4, 6)	4	(2, 6)	<0.001
Laboratory data									
BNP, pg/mL	243	(114, 499)	360	(168, 592)	196	(85, 398)	125	(82, 208)	<0.001
Albumin, g/dL	3.6	(3.3, 3.8)	3.5	(3.1, 3.8)	3.6	(3.3, 3.9)	3.6	(3.3, 3.8)	0.004
Hemoglobin, g/dL	11.3	(10.3, 12.6)	11.1	(10.1, 12.2)	11.7	(10.5, 12.9)	12.1	(10.8, 13.5)	<0.001
Uric acid, mg/dL	6.0	(4.9, 7.3)	6.3	(4.8, 7.5)	5.9	(5.0, 7.3)	5.7	(4.6, 6.5)	0.236
Creatinine, mg/dL	1.02	(0.80, 1.40)	1.10	(0.80, 1.46)	1.02	(0.79, 1.41)	0.88	(0.65, 1.14)	0.045
eGFRcre, mL/min/1.73m^*2*^	47.6	(33.7, 60.6)	45.2	(32.6, 59.3)	48.4	(35.3, 59.4)	57.9	(45.4, 68.0)	0.016
Sodium, mEq/L	140	(137, 142)	139	(136, 141)	141	(138, 142)	141	(139, 142)	<0.001
Total lymphocyte counts, /μL	1299	(943, 1682)	1270	(925, 1606)	1343	(976, 1748)	1350	(1008, 1729)	0.295
Medication,* N* (%)									
β-Blocker	304	(73)	162	(75)	121	(72)	21	(62)	0.288
ACE-I or ARB	212	(51)	104	(48)	90	(54)	18	(53)	0.525
MRA	220	(53)	129	(59)	79	(47)	12	(35)	0.006
Loop diuretics	291	(70)	161	(74)	110	(65)	20	(59)	0.069
Statin	204	(49)	102	(47)	81	(48)	21	(62)	0.274
XO inhibitor	129	(31)	70	(32)	54	(32)	5	(15)	0.106
MNA-SF score, points	7	(6, 9)	6	(5, 7)	9	(8, 10)	12	(12, 13)	<0.001
EI, kcal/day	1481	(1232, 1600)	1353	(1091, 1550)	1550	(1400, 1600)	1580	(1429, 1600)	<0.001
Per body weight, kcal/kg/day									
EI per actual body weight	27.0	± 7.4	27.3	± 8.5	26.7	± 6.2	26.4	± 5.3	0.670
EI per standard body weight	26.7	(22.2, 29.5)	24.8	(19.7, 28.5)	27.9	(24.9, 30.1)	29.3	(26.4, 30.7)	<0.001
EI per target body weight	24.1	(20.3, 26.7)	22.2	(17.8, 25.6)	25.0	(22.6, 27.3)	26.1	(23.7, 28.0)	<0.001
Daily protein intake, g/kg/day	1.13	± 0.34	1.14	± 0.38	1.12	± 0.29	1.15	± 0.29	0.887
All-cause death,* N* (%)	110	(26)	78	(36)	31	(18)	1	(3)	<0.001
Heart failure	51		36		14		1		0.968
Infection	22		15		7		0		
Cancer	12		9		3		0		
Others	25		18		7		0		

Data are presented as mean ± standard deviation of the mean, median (interquartile range, 25th, 75th percentile), or number (with percentage). *N* number of patients for whom the parameter was available

*BMI* body mass index, *NYHA* New York Heart Association, *LVEF* left ventricular ejection fraction, *HF* heart failure, *ICD* implantable cardioverter defibrillator, *CRT-P* cardiac resynchronization therapy pacemaker, *CRT-D* cardiac resynchronization therapy defibrillator, *BNP* B-type natriuretic peptide, *eGFRcre* creatinine-based estimated glomerular filtration rate, *ACE-I* angiotensin converting enzyme inhibitor, *ARB* angiotensin receptor-blocker, *MRA* mineralocorticoid receptor antagonist, *XO* xanthine oxidase, *MNA-SF* mini nutritional assessment short form, *EI* daily energy intake

### Relationships between MNA-SF score, energy intake, and clinical parameters

**Fig. 2:**
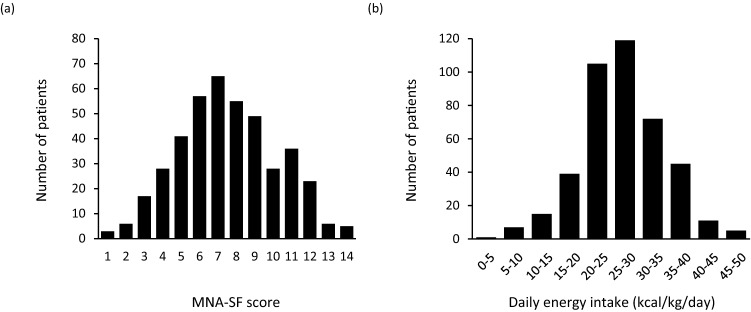
Distribution of MNA-SF scores (**a**) and daily energy intake levels (**b**). Energy intake (kcal/kg/day) was calculated by dividing energy intake per day by actual body weight. *MNA-SF* the Mini Nutritional Assessment Short Form

The distribution of MNA-SF scores is shown in Fig. 2a. The median MNA-SF score was 7 points (IQR 6–9 points), and 52% and 40% of the patients were classified as malnutrition and at risk of malnutrition, respectively, resulting in limited number of patients with normal nutritional status (Table 1). The high prevalence of malnutrition and at risk of malnutrition assessed by MNA-SF may be due to the low BMI in this Japanese cohort, leading to possible overestimation of the prevalence of malnutrition. For this reason, we divided patients into tertile groups (Low MNA-SF, ≤ 6; Mid MNA-SF, 7–9; High MNA-SF, ≥ 10) according to MNA-SF scores in addition to the standard categorization of nutritional status by MNA-SF scores to analyze the impact of MNA-SF scores on all-cause mortality in HF patients. As shown in Table 2, there were no significant differences in age, percentage of females, and LVEF among the groups. As MNA-SF scores decreased, BMI, systolic blood pressure, Barthel index scores, and concentrations of albumin, hemoglobin and sodium tended to decrease, whereas heart rate, prevalence of history of HF hospitalization, proportions of patients with diabetes and cachexia, Charlson Comorbidity Index, BNP, and proportion of patients receiving loop diuretics and mineralocorticoid receptor antagonists tended to increase. Amount of daily energy intake tended to be lower in the Low MNA-SF group. Similar trends were found when HF patients were classified into normal nutritional status, at risk of malnutrition, and malnourished, except that proportion of patients with HFrEF and NYHA functional class IV tended to be higher and eGFRcre tended to be lower as nutritional status worsened (Table 1).

**Table 2:** Baseline characteristics according to tertile groups of MNA-SF scores

Variables	All		MNA-SF score						
			Low		Mid		High		*p* value
			≤ 6 points		7–9 points		≥ 10 points		
	*N* = 419		*N* = 152		*N* = 169		*N* = 98		
Age, years	78	(72, 83)	77	(71, 83)	78	(72, 84)	78	(71, 82)	0.538
≥ 75 years,* N* (%)	257	(61)	91	(60)	106	(63)	60	(61)	0.871
Female,* N* (%)	204	(49)	76	(50)	79	(47)	49	(50)	0.808
Height, cm	157	± 9	158	± 9	156	± 9	156	± 9	0.405
Body weight, kg	51.6	(44.5, 59.8)	46.2	(40.6, 52.1)	53.4	(47.2, 61.2)	58.2	(51.1, 65.1)	<0.001
BMI, kg/m^*2*^	21.1	(18.6, 23.4)	18.3	(16.8, 20.7)	21.9	(20.1, 24.0)	23.6	(21.1, 26.6)	<0.001
Heart rate, bpm	68	(60, 75)	70	(62, 80)	67	(60, 74)	65	(60, 72)	<0.001
Systolic blood pressure, mmHg	116	(103, 129)	110	(100, 123)	118	(105, 130)	120	(108, 131)	0.001
NYHA functional class,* N* (%)									<0.001
I	23	(6)	5	(3)	8	(5)	10	(10)	
II	245	(59)	82	(54)	106	(63)	57	(58)	
III	129	(31)	48	(32)	51	(30)	30	(31)	
IV	22	(5)	17	(11)	4	(2)	1	(1)	
LVEF, %	47.6	(34.1, 62.7)	45.6	(32.1, 61.0)	48.2	(35.7, 63.2)	50.4	(35.5, 63.4)	0.277
< 40%,* N* (%)	142	(34)	61	(40)	53	(31)	28	(29)	0.113
Smoking history,* N* (%)	130	(31)	45	(30)	48	(28)	37	(38)	0.252
Barthel Index score, points	85	(75, 90)	80	(65, 85)	85	(75, 90)	90	(80, 95)	<0.001
History of HF hospitalization,* N* (%)	207	(51)	89	(59)	78	(46)	40	(41)	0.013
Etiology,* N* (%)									0.083
Valvular heart disease	140	(33)	47	(31)	55	(33)	38	(39)	
Cardiomyopathy	114	(27)	36	(24)	43	(25)	35	(36)	
Ischemic	87	(21)	36	(24)	38	(22)	13	(13)	
Device,* N* (%)									0.171
Pacemaker	62	(15)	27	(18)	25	(15)	10	(10)	
ICD	31	(8)	5	(3)	15	(9)	11	(11)	
CRT-P or CRT-D	28	(7)	12	(8)	9	(5)	7	(7)	
Comorbidity,* N* (%)									
Hypertension	299	(71)	97	(64)	133	(79)	69	(70)	0.013
Diabetes mellitus	190	(45)	80	(53)	77	(46)	33	(34)	0.013
Dyslipidemia	226	(54)	74	(49)	97	(57)	55	(56)	0.261
Chronic kidney disease	312	(75)	112	(74)	134	(79)	66	(67)	0.094
Atrial fibrillation	189	(45)	77	(51)	71	(42)	41	(42)	0.227
Chronic pulmonary disease	100	(24)	43	(28)	41	(24)	16	(16)	0.095
History of cancer	108	(26)	39	(26)	48	(28)	21	(21)	0.454
Orthopedic disorder	132	(32)	51	(34)	48	(28)	33	(34)	0.532
Cachexia	41	(10)	26	(13)	14	(8)	1	(1)	<0.001
Charlson Comorbidity Index, *points*	5	(4, 7)	5	(4, 7)	5	(4, 7)	4	(3, 6)	<0.001
Laboratory data									
BNP, pg/mL	243	(114, 499)	388	(178, 623)	243	(110, 467)	147	(80, 320)	<0.001
Albumin, g/dL	3.6	(3.3, 3.8)	3.5	(3.1, 3.7)	3.6	(3.3, 3.9)	3.6	(3.4, 3.8)	0.005
Hemoglobin, g/dL	11.3	(10.3, 12.6)	11.0	(10.0, 12.0)	11.5	(10.4, 12.7)	11.7	(10.6, 13.1)	0.002
Uric acid, mg/dL	6.0	(4.9, 7.3)	6.1	(4.8, 7.5)	6.2	(5.2, 7.4)	5.8	(4.9, 6.9)	0.203
Creatinine, mg/dL	1.02	(0.80, 1.40)	1.06	(0.80, 1.34)	1.08	(0.80, 1.58)	0.97	(0.76, 1.22)	0.120
eGFRcre, mL/min/1.73m^*2*^	47.6	(33.7, 60.6)	46.8	(33.0, 61.7)	46.3	(31.6, 58.8)	51.8	(39.5, 63.8)	0.082
Sodium, mEq/L	140	(137, 142)	138	(135, 141)	140	(137, 142)	141	(139, 142)	<0.001
Total lymphocyte counts, /μL	1299	(943, 1682)	1230	(912, 1568)	1360	(966, 1755)	1302	(998, 1639)	0.151
Medication,* N* (%)									
β-Blocker	304	(73)	111	(73)	123	(73)	70	(71)	0.959
ACE-I or ARB	212	(51)	70	(46)	88	(52)	54	(55)	0.333
MRA	220	(53)	94	(62)	87	(51)	39	(40)	0.003
Loop diuretics	291	(70)	115	(76)	119	(70)	57	(58)	0.013
Statin	204	(49)	71	(47)	83	(49)	50	(51)	0.793
XO inhibitor	129	(31)	49	(32)	56	(33)	24	(25)	0.300
MNA-SF score, points	7	(6, 9)	5	(4, 6)	8	(7, 9)	11	(10, 12)	<0.001
EI, kcal/day	1481	(1232, 1600)	1350	(1058, 1550)	1540	(1267, 1600)	1551	(1400, 1600)	<0.001
per body weight, kcal/kg/day									
EI per actual body weight	27.0	± 7.4	27.4	± 8.8	26.9	± 7.2	26.5	± 5.2	0.592
EI per standard body weight	26.7	(22.2, 29.5)	24.4	(19.0, 27.9)	27.2	(23.2, 29.7)	28.5	(26.0, 30.7)	<0.001
EI per target body weight	24.1	(20.3, 26.7)	21.6	(17.0, 25.2)	24.4	(21.2, 26.8)	25.3	(23.7, 27.6)	<0.001
Daily protein intake, g/kg/day	1.13	± 0.34	1.15	± 0.38	1.11	± 0.33	1.14	± 0.26	0.574
All-cause death,* N* (%)	110	(26)	61	(40)	39	(23)	10	(10)	<0.001
Heart failure	51		29		17		5		0.912
Infection	22		10		10		2		
Cancer	12		6		5		1		
Others	25		16		7		2		

Data are presented as mean ± standard deviation of the mean, median (interquartile range, 25th, 75th percentile), or number (with percentage). *N* number of patients for whom the parameter was available

*BMI* body mass index, *NYHA* New York Heart Association, *LVEF* left ventricular ejection fraction, *HF* heart failure, *ICD* implantable cardioverter defibrillator, *CRT-P* cardiac resynchronization therapy pacemaker, *CRT-D* cardiac resynchronization therapy defibrillator, *BNP* B-type natriuretic peptide, *eGFRcre* creatinine-based estimated glomerular filtration rate, *ACE-I* angiotensin converting enzyme inhibitor, *ARB* angiotensin receptor-blocker, *MRA* mineralocorticoid receptor antagonist, *XO* xanthine oxidase, *MNA-SF* mini nutritional assessment short form, *EI* daily energy intake

The distribution of amounts of daily energy intake per actual body weight is shown in Fig. 2b. The median daily energy intake per day was 1481 kcal/day (IQR 1232–1600), and the mean energy intake per actual body weight was 27.0 ± 7.4 kcal/kg/day. As shown in Table 3, when patients were divided into tertile groups (low EI ≤ 23.9 kcal/kg/day; mid EI 24.0–29.8; high EI ≥ 29.9) according to daily energy intake per actual body weight, there were no significant differences in age, percentage of females, heart rate, systolic blood pressure, LVEF, NYHA functional class, prevalence of history of HF hospitalization, and BNP level among the groups. As energy intake per actual weight decreased, body weight, BMI, Charlson Comorbidity Index, and levels of creatinine and uric acid tended to increase.

**Table 3:** Baseline characteristics according to tertile groups of daily energy intake

Variables	All		EI (per actual body weight)						
			Low		Mid		High		*p* value
			≤ 23.9 kcal/kg/day		24.0–29.8 kcal/kg/day		≥ 29.9 kcal/kg/day		
	*N* = 419		*N* = 139		*N* = 141		*N* = 139		
Age, years	78	(72, 83)	78	(72, 83)	77	(72, 82)	77	(71, 84)	0.665
≥ 75 years,* N* (%)	257	(61)	86	(62)	88	(62)	83	(60)	0.887
Female,* N* (%)	204	(49)	73	(53)	84	(60)	58	(42)	0.011
Height, cm	157	± 9	158	± 9	158	± 9	154	± 9	<0.001
Body weight, kg	51.6	(44.5, 59.8)	58.8	(47.8, 67.0)	55.3	(50.9, 60.4)	45.1	(40.6, 50.0)	<0.001
BMI, kg/m^*2*^	21.1	(18.6, 23.4)	22.7	(19.9, 25.5)	22.2	(20.4, 23.9)	19.0	(17.3, 20.5)	<0.001
Heart rate, bpm	68	(60, 75)	68	(60, 75)	68	(60, 76)	68	(61, 75)	0.886
Systolic blood pressure, mmHg	116	(103, 129)	115	(104, 128)	117	(102, 129)	116	(103, 129)	0.952
NYHA functional class,* N* (%)									0.095
I	23	(6)	5	(4)	9	(6)	9	(6)	
II	245	(59)	81	(58)	74	(52)	90	(65)	
III	129	(31)	41	(30)	52	(37)	36	(26)	
IV	22	(5)	12	(9)	6	(4)	4	(3)	
LVEF, %	47.6	(34.1, 62.7)	47.5	(36.5, 61.1)	48.6	(34.1, 63.3)	46.5	(32.3, 63.3)	0.841
< 40%,* N* (%)	142	(34)	44	(32)	46	(33)	52	(37)	0.554
Smoking history,* N* (%)	130	(31)	45	(32)	49	(35)	36	(26)	0.254
Barthel Index score, points	85	(75, 90)	80	(65, 90)	85	(80, 95)	85	(75, 90)	0.001
History of HF hospitalization,* N* (%)	207	(51)	67	(48)	72	(51)	68	(49)	0.883
Etiology,* N* (%)									0.417
Valvular heart disease	140	(33)	38	(27)	52	(37)	50	(36)	
Cardiomyopathy	114	(27)	37	(27)	41	(29)	36	(26)	
Ischemic	87	(21)	32	(23)	28	(20)	27	(19)	
Device,* N* (%)									0.093
Pacemaker	62	(15)	26	(19)	17	(12)	19	(14)	
ICD	31	(8)	7	(5)	15	(11)	9	(6)	
CRT-P or CRT-D	28	(7)	11	(8)	4	(3)	13	(9)	
Comorbidity,* N* (%)									
Hypertension	299	(71)	105	(76)	101	(72)	93	(67)	0.281
Diabetes mellitus	190	(45)	64	(46)	63	(45)	63	(45)	0.974
Dyslipidemia	226	(54)	80	(58)	81	(57)	65	(47)	0.116
Chronic kidney disease	312	(75)	115	(83)	101	(72)	96	(69)	0.021
Atrial fibrillation	189	(45)	70	(50)	62	(44)	57	(41)	0.277
Chronic pulmonary disease	100	(24)	34	(24)	34	(24)	32	(23)	0.958
History of cancer	108	(26)	36	(26)	40	(28)	32	(23)	0.592
Orthopedic disorder	132	(32)	48	(35)	39	(28)	45	(32)	0.448
Cachexia	41	(10)	20	(14)	11	(8)	10	(7)	0.081
Charlson Comorbidity Index, points	5	(4, 7)	6	(4, 7)	5	(4, 6)	5	(3, 7)	0.039
Laboratory data									
BNP, pg/mL	243	(114, 499)	290	(119, 519)	238	(102, 461)	234	(117, 509)	0.404
Albumin, g/dL	3.6	(3.3, 3.8)	3.6	(3.3, 3.9)	3.6	(3.3, 3.9)	3.5	(3.2, 3.8)	0.399
Hemoglobin, g/dL	11.3	(10.3, 12.6)	11.4	(10.3, 12.8)	11.7	(10.4, 12.7)	11.0	(10.2, 12.0)	0.049
Uric acid, mg/dL	6.0	(4.9, 7.3)	6.6	(5.4, 7.6)	6.0	(4.9, 7.2)	5.5	(4.5, 6.9)	<0.001
Creatinine, mg/dL	1.02	(0.80, 1.40)	1.11	(0.89, 1.45)	1.02	(0.81, 1.51)	0.94	(0.71, 1.30)	0.011
eGFRcre, mL/min/1.73m^*2*^	47.6	(33.7, 60.6)	44.8	(32.7, 57.1)	50.8	(33.9, 61.8)	50.6	(35.7, 64.5)	0.036
Sodium, mEq/L	140	(137, 142)	139	(136, 142)	140	(138, 142)	139	(136, 141)	0.007
Total lymphocyte counts, /μL	1299	(943, 1682)	1290	(994, 1645)	1370	(981, 1775)	1212	(886, 1605)	0.158
Medication,* N* (%)									
β Blocker	304	(73)	100	(72)	99	(70)	105	(76)	0.596
ACE-I or ARB	212	(51)	70	(50)	79	(56)	63	(45)	0.201
MRA	220	(53)	72	(52)	71	(50)	77	(55)	0.686
Loop diuretics	291	(70)	96	(69)	99	(70)	96	(69)	0.971
Statin	204	(49)	66	(47)	79	(56)	59	(42)	0.071
XO inhibitor	129	(31)	46	(33)	39	(28)	44	(32)	0.594
MNA-SF score, points	7	(6, 9)	7	(6, 9)	8	(6, 10)	7	(5, 9)	0.002
Malnutrition (≤ 7),* N* (%)	217	(52)	73	(53)	60	(43)	84	(60)	0.039
At risk (6–11),* N* (%)	168	(40)	57	(41)	65	(46)	46	(33)	
Normal (≥ 12),* N* (%)	34	(8)	9	(6)	16	(11)	9	(6)	
EI, kcal/day	1481	(1232, 1600)	1143	(817, 1400)	1550	(1350, 1600)	1550	(1400, 1600)	<0.001
Per body weight, kcal/kg/day									
EI per actual body weight	27.0	± 7.4	19.1	± 4.3	26.8	± 1.8	35.0	± 4.3	<0.001
EI per standard body weigh	26.7	(22.2, 29.5)	20.8	(16.3, 24.4)	27.2	(24.8, 29.3)	29.6	(27.5, 31.9)	<0.001
EI per target body weight	24.1	(20.3, 26.7)	18.7	(14.3, 21.9)	24.8	(22.2, 26.2)	26.8	(24.6, 28.9)	<0.001
Daily protein intake, g/kg/day	1.13	± 0.34	0.82	± 0.24	1.15	± 0.17	1.43	± 0.27	<0.001
All-cause death,* N* (%)	110	(26)	47	(34)	33	(23)	30	(22)	0.044
Heart failure	51		22		16		13		0.620
Infection	22		11		6		5		
Cancer	12		2		5		5		
Others	25		12		6		7		

Data are presented as mean ± standard deviation of the mean, median (interquartile range, 25th, 75th percentile), or number (with percentage).* N* number of patients for whom the parameter was available

*BMI* body mass index, *NYHA* New York Heart Association, *LVEF* left ventricular ejection fraction, *HF* heart failure, *ICD* implantable cardioverter defibrillator, *CRT-P* cardiac resynchronization therapy pacemaker, *CRT-D* cardiac resynchronization therapy defibrillator, *BNP* B-type natriuretic peptide, *eGFRcre* creatinine-based estimated glomerular filtration rate, *ACE-I* angiotensin converting enzyme inhibitor, *ARB* angiotensin receptor blocker, *MRA* mineralocorticoid receptor antagonist, *XO* xanthine oxidase, *MNA-SF* mini nutritional assessment short form, *EI* daily energy intake

### Impact of MNA-SF score and energy intake on all-cause mortality in HF patients

**Fig. 3:**
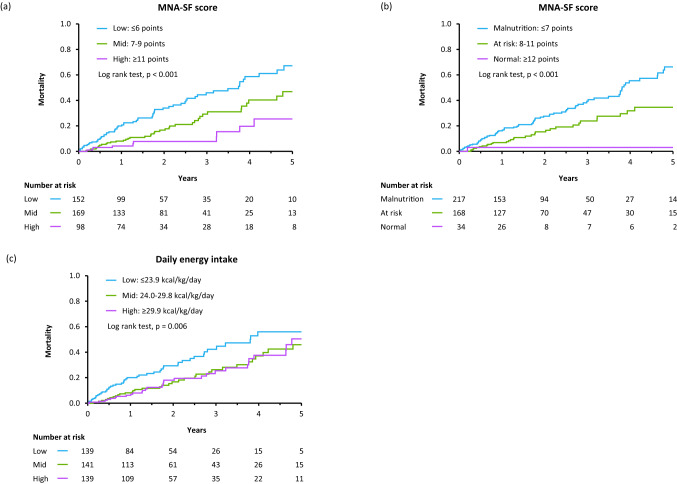
Kaplan-Meier survival curves showing impact of nutritional status (**a**–**b**) and energy intake (**c**) on all-cause mortality in HF patients. **a** HF patients were subdivided into tertile groups (low MNA-SF, ≤ 6; mid MNA-SF, 7–9; high MNA-SF, ≥ 10) according to MNA-SF scores. **b** HF patients were subdivided according to the standard categorization of nutritional status by MNA-SF scores: normal nutritional status, 12–14; at risk of malnutrition, 8–11; malnutrition, 0–7. **c** HF patients were subdivided into tertile groups (low EI, ≤ 23.9 kcal/kg/day; mid EI, 24.0–29.8; high EI, ≥ 29.9) according to energy intake per actual body weight. *MNA-SF* the Mini Nutritional Assessment Short Form

During a median 1.52-year period (IQR 0.96–2.94 years), 110 patients (26%) died (51 patients from HF-related causes; 22 patients from infection; 12 patients from cancer). As shown in Fig. 3, Kaplan–Meier survival curves showed that all-cause mortality increased as nutritional status worsened, whereas all-cause mortality rate was higher in patients with Low EI among the EI groups.

**Fig. 4:**
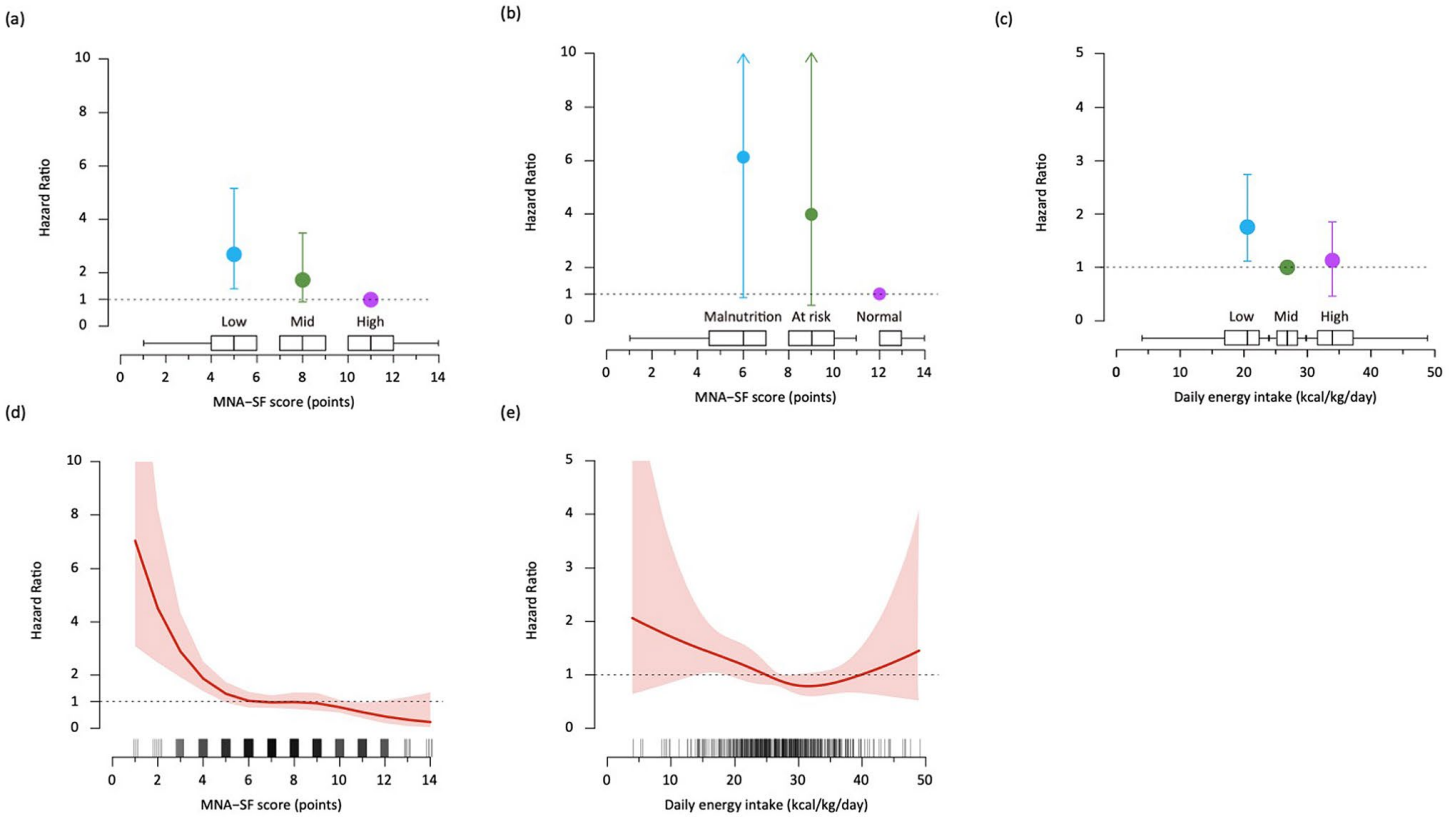
Forest plots of hazard ratios and 95% confidence intervals for the all-cause mortality according to nutritional status (**a**, **b**) and energy intake (**c**) in HF patients. All analyses were adjusted for age, gender, log BNP, NYHA functional class, Charlson Comorbidity Index score, history of HF hospitalization, and cachexia. **a** HF patients were subdivided into tertile groups (low MNA-SF, ≤ 6; mid MNA-SF, 7–9; high MNA-SF, ≥ 10) according to MNA-SF scores. **b** HF patients were subdivided according to the standard categorization of nutritional status by MNA-SF scores: normal nutritional status, 12–14; at risk of malnutrition, 8–11; malnutrition, 0–7. **c** HF patients were subdivided into tertile groups (low EI, ≤ 23.9 kcal/kg/day; mid EI, 24.0–29.8; high EI, ≥ 29.9) according to energy intake per actual body weight. **d**, **e** Adjusted dose-dependent association of energy intake and all-cause mortality in HF patients. The dotted line represents a hazard ratio of 1.0, the red line represents hazard ratios, and the light red areas represent 95% confidence intervals. Rug plots are shown along the x-axes of the graphs to depict the distributions of daily energy intake levels. All analyses were adjusted for age, gender, log BNP, NYHA functional class, Charlson Comorbidity Index score, history of HF hospitalization, and cachexia. *MNA-SF* the Mini Nutritional Assessment Short Form, *EI* daily energy intake, *BNP* B-type natriuretic peptide, *NYHA* New York Heart Association, *HF* heart failure

In multivariate Cox-proportional hazard analyses with adjustment for potential confounders (Model 1 consisting of age, sex, and BMI; Model 2 consisting of age, sex, and BNP; and Model 3 consisting of Model 2, NYHA functional class, Charlson Comorbidity Index, history of HF hospitalization, and cachexia), hazard ratios for the all-cause mortality were significantly higher in the low or mid MNA-SF group than in the high MNA-SF group as a reference (Table 4, Fig. 4a). After adjustment for daily energy intake per actual body weight in addition to Model 3, the significant association between low or mid MNA-SF score and all-cause mortality was preserved (Table 4). Similar results were found when HF patients were classified according to the standard categorization of nutritional status (Table 4, Fig. 4b).

**Table 4:** Impact of MNA-SF score and daily energy intake on all-cause mortality in HF patients

Models	Wald’s χ^2^	*p* value	MNA-SF score (standard category)					
			Malnutrition		At risk		Normal (Reference)	
			≤ 7 points		8–11 points		≥ 12 points	
			*N* = 217		*N* = 168		*N* = 34	
			HR	(95% CI)	HR	(95% CI)	HR	(95% CI)
Unadjusted model	16.94	< 0.001	10.70 (1.49–76.96)		5.07 (0.69–37.17)		1.00	
Adjusted model 1	11.96	0.003	9.02 (1.25–65.18)		4.85 (0.66–35.56)		1.00	
Adjusted model 2	8.80	0.012	6.87 (0.94–50.01)		4.03 (0.55–29.69)		1.00	
Adjusted model 3	6.00	0.049	6.04 (0.81–44.91)		3.99 (0.53–29.88)		1.00	
+ EI per actual body weight (continuous)	6.55	0.038	6.18 (0.83–45.95)		3.95 (0.53–29.62)		1.00	
+ EI per actual body weight (tertile)	6.65	0.036	6.11 (0.81–45.84)		3.86 (0.51–29.09)		1.00	

Models	Wald’s χ^2^	*p* value	MNA-SF score (tertile)		
			Malnutrition	At risk	Normal (Reference)
			Low	Mid	High (Reference)
			≤ 6 points	7–10 points	≥ 11 points
			*N* = 152	*N* = 169	*N* = 98
			HR (95% CI)	HR (95% CI)	HR (95% CI)
Unadjusted model	23.92	< 0.001	4.27 (2.19–8.34)	2.15 (1.07–4.32)	1.00
Adjusted model 1	17.13	< 0.001	3.64 (1.85–7.18)	2.06 (1.03–4.13)	1.00
Adjusted model 2	14.03	< 0.001	3.12 (1.57–6.18)	1.80 (0.89—3.63)	1.00
Adjusted model 3	8.76	0.013	2.58 (1.29–5.16)	1.70 (0.84–3.44)	1.cxs00
+ EI per actual body weight (continuous)	9.78	0.008	2.69 (1.34–5.39)	1.70 (0.84–3.46)	1.00
+ EI per actual body weight (tertile)	10.09	0.006	2.70 (1.34–5.43)	1.67 (0.82–3.39)	1.00

Models	Wald’s χ^2^	*p* value	EI (per actual body weight)		
			Low	Mid (Reference)	High
			≤ 23.9 kcal/kg/day	24.0–29.8 kcal/kg/day	≥ 29.9 kcal/kg/day
			*N* = 139	*N* = 141	*N* = 139
			HR (95% CI)	HR	HR (95% CI)
Unadjusted model	10.01	0.007	1.84 (1.18–2.88)	1.00	1.00 (0.61–1.63)
Adjusted model 1	9.34	0.009	1.77 (1.13–2.77)	1.00	0.95 (0.58–1.57)
Adjusted model 2	8.82	0.012	1.74 (1.11–2.72)	1.00	0.94 (0.57–1.56)
Adjusted model 3	5.95	0.051	1.71 (1.07–2.73)	1.00	1.11 (0.67–1.83)
+ MNA-SF score (ontinuous)	7.58	0.023	1.71 (1.07–2.71)	1.00	0.96 (0.58–1.60)
+ MNA-SF score (standard category)	6.57	0.037	1.69 (1.06–2.68)	1.00	1.01 (0.61–1.68)
+ MNA-SF score (tertile)	7.35	0.025	1.72 (1.08–2.73)	1.00	0.99 (0.59–1.64)

Models	Wald’s χ^2^	*p* value	EI (per standard body weight)		
			Low	Mid (Reference)	High
			≤ 24.0 kcal/kg/day	24.1–28.5 kcal/kg/day	≥ 28.6 kcal/kg/day
			*N* = 139	*N* = 140	*N* = 140
			HR (95% CI)	HR	HR (95% CI)
Unadjusted model	22.51	< 0.001	1.80 (1.18–2.75)	1.00	0.54 (0.31–0.94)
Adjusted model 1	18.78	< 0.001	1.70 (1.11–2.60)	1.00	0.55 (0.31–0.96)
Adjusted model 2	18.03	< 0.001	1.61 (1.05–2.47)	1.00	0.51 (0.29–0.91)
Adjusted model 3	13.49	0.001	1.47 (0.96–2.28)	1.00	0.54 (0.31–0.96)
+ MNA-SF score (continuous)	5.80	0.055	1.30 (0.83–2.01)	1.00	0.65 (0.36–1.15)
+ MNA-SF score (standard category)	8.54	0.014	1.41 (0.90–2.19)	1.00	0.62 (0.35–1.10)
+ MNA-SF score (tertile)	7.66	0.022	1.36 (0.88–2.11)	1.00	0.62 (0.35–1.11)

Models	Wald’s χ^2^	*p* value	EI (per target body weight)		
			Low	Mid (Reference)	High
			≤ 21.7 kcal/kg/day	21.8–25.7 kcal/kg/day	≥ 25.8 kcal/kg/day
			*N* = 139	*N* = 140	*N* = 140
			HR (95% CI)	HR	HR (95% CI)
Unadjusted model	23.71	< 0.001	2.18 (1.41–3.39)	1.00	0.74 (0.43–1.27)
Adjusted model 1	18.67	< 0.001	1.95 (1.25–3.05)	1.00	0.71 (0.41–1.24)
Adjusted model 2	18.32	< 0.001	1.94 (1.24–3.04)	1.00	0.71 (0.41–1.24)
Adjusted model 3	10.11	0.006	1.67 (1.06–2.63)	1.00	0.77 (0.45–1.33)
+ MNA-SF score (continuous)	3.78	0.151	1.40 (0.88–2.24)	1.00	0.86 (0.49–1.49)
+ MNA-SF score (standard category)	6.97	0.031	1.60 (1.00–2.53)	1.00	0.84 (0.48–1.46)
+ MNA-SF score (tertile)	5.92	0.052	1.52 (0.96–2.41)	1.00	0.83 (0.48–1.45)

Adjusted models: Model 1 including age, sex and BMI; Model 2 including age, sex and log BNP; Model 3 including age, sex, log BNP, NYHA functional class, Charlson Comorbidity Index score, history of HF hospitalization and cachexia. Abbreviations: HR, hazard ratio; CI, confidence interval; MNA-SF, mini nutritional assessment short form; EI, daily energy intake; BNP, B-type natriuretic peptide; NYHA, New York Heart Association; HF, heart failure

Analyses of daily energy intake per actual body weight showed that hazard ratios for all-cause mortality were significantly higher in the Low EI group than in the Mid EI group as a reference after adjustments for Model 1 and Model 2, respectively, and tended to be higher in the Low EI group than in the Mid EI group as a reference after adjustments for Model 3 (*p* = 0.051, Table 4, Fig. 4c). An independent association between daily energy intake per actual body weight and all-cause mortality remained in the model adjusted for MNA-SF score in addition to Model 3 (Table 4). The hazard ratio in the High EI group paradoxically tended to higher than in the Mid EI group (Table 4, Fig. 4c). Analyses of daily energy intake per standard body weight and energy intake per target body weight yielded almost similar results (Table 4).

To further examine the impact of nutritional status and energy intake on the all-cause mortality, we examined a fully adjusted dose-dependent association of MNA-SF scores and daily energy intake per actual body weight with all-cause mortality by use of a Cox regression model with a restricted cubic spline function with four knots. As expected, risk of all-cause mortality increased as MNA-SF scores decreased (Fig. 4d). The results shown in Fig. 4e indicated that risk of all-cause mortality was low among HF patients who consumed 25.0–40.0 kcal/kg/day of energy (hazard ratio < 1.0) and HF patients who consumed 31.5 kcal/kg/day of energy had the lowest risk of all-cause mortality.

### Impact of MNA-SF score and energy intake on prediction of all-cause mortality in HF patients

Based on the results showing that MNA-SF score and energy intake are independent predictors of all-cause mortality in HF patients, we examined whether combined assessment of MNA-SF and energy intake improves the accuracy of prediction of mortality in HF patients by calculating C-index, cNRI, and IDI. Addition of daily energy intake per actual body weight to MNA-SF score tended to improve the C-index (MNA-SF, 0.690 [IQR 0.631–0.744]; MNA-SF + energy intake, 0.708 [IQR 0.647–0.762]), and it significantly improved cNRI (0.220, *p* = 0.048) and IDI (0.018, *p* = 0.011, Table 5). In addition, inclusion of MNA-SF and daily energy intake per actual body weight to each baseline model significantly improved both cNRI and IDI in addition to the C-index (Table 5). These results suggest an improvement in prediction of the all-cause mortality in HF patients by addition of both MNA-SF and energy intake to the baseline models compared with baseline models alone.

**Table 5:** Impact of MNA-SF score and energy intake on prediction of all-cause mortality in HF patients

Models	C-index	(95% CI)	LLR improvement from base model	p value	cNRI	(95% CI)	*p* value	IDI	(95% CI)	*p* value
MNA-SF score	0.690	(0.631–0.744)		Ref.			Ref.			Ref.
+EI per actual body weight	0.708	(0.647–0.762)	− 1.090	0.140	0.220	(0.004–0.436)	0.048	0.018	(0.004–0.032)	0.011
Model 1	0.616	(0.549–0.679)		Ref.			Ref.			Ref.
+ MNA-SF score + EI per actual body weight	0.726	(0.665–0.779)	− 5.775	< 0.001	0.478	(0.267–0.689)	< 0.001	0.076	(0.048–0.014)	< 0.001
Model 2	0.708	(0.649–0.761)		Ref.			Ref.			Ref.
+ MNA-SF score + EI per actual body weight	0.752	(0.696–0.801)	− 2.364	0.030	0.499	(0.287–0.710)	< 0.001	0.056	(0.030–0.083)	< 0.001
Model 3	0.745	(0.686–0.795)		Ref.			Ref.			Ref.
+ MNA-SF score + EI per actual body weight	0.771	(0.716–0.819)	− 1.789	0.059	0.355	(0.140–0.569)	0.003	0.029	(0.010–0.049)	0.003

Adjusted models: Model 1 including age, sex and BMI; Model 2 including age, sex and log BNP; Model 3 including age, sex, log BNP, NYHA functional class, Charlson Comorbidity Index score, history of HF hospitalization and cachexia

*CI* confidence interval, *MNA-SF* mini nutritional assessment short form, *EI* daily energy intake, *LLR* log-likelihood ratio, *cNRI* continuous net reclassification improvement, *IDI* integrated discrimination improvement, *BNP* B-type natriuretic peptide, *NYHA* New York Heart Association, *HF* heart failure

### Impact of energy intake on prediction of all-cause mortality in HF patients with and those without Low MNA-SF

**Fig. 5:**
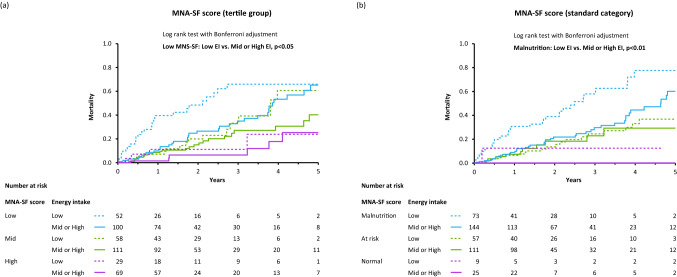
Kaplan-Meier survival curves showing impact of energy intake on the all-cause mortality in HF patients subdivided according to tertile groups of MNA-SF scores (**a**) and the standard categorization of nutritional status by MNA-SF scores (**b**). *MNA-SF* the Mini Nutritional Assessment Short Form, *EI* total energy intake

In a subgroup with Low MNA-SF scores and malnutrition, patients with Low EI had a significantly higher all-cause mortality rate than did patients without Low EI (Fig. 5). On the other hand, Kaplan–Meier curves of all-cause mortality did not differ between patients with and those without Low EI in subgroups of patients without Low MNA-SF scores and malnutrition (Fig. 5).

## Discussion

There are two salient findings in the present study. First, we found that daily energy intake during hospitalization was an independent predictor of all-cause mortality after discharge in HF patients after adjustment for nutritional status and known prognostic markers including BNP and cachexia. Second, addition of daily energy intake to established predictors of the prognosis of HF improves risk stratification of elderly patients with HF. Addition of daily energy intake to MNA-SF score improved the predictive ability of MNA-SF alone, and low daily energy intake could detect higher risk patients even in a subgroup of HF patients with malnutrition. These findings suggest that assessment of energy intake is important in risk stratification for mortality of elderly HF patients, especially those with malnutrition and in planning comprehensive therapy for HF.

Since energy intake and nutritional status after discharge were not recorded for performing longitudinal analyses in the present study, we could not determine whether level of energy intake is a modifiable factor that causally determines outcomes of HF or a marker of severity of HF. Improvement of energy intake by optimal HF therapy during hospitalization is likely to contribute to restoration of nutritional status and muscle mass, leading to reduction in mortality, since nutritional intervention including macronutrients supplementation has been shown to have favorable effects on body composition, exercise capacity and prognosis [24–26]. On the other hand, cachexia is a hallmark in the advanced stage of HF and it is an independent predictor of mortality [12, 27, 28]. Cachexia-induced systemic inflammation exaggerates malnutrition, i.e., chronic disease-related malnutrition with inflammation [8], which is frequently complicated with anorexia, appetite loss, nausea, and taste disorder [29]. Thus, low energy intake at the time of discharge might serve as a marker of cachexia, leading to poor outcome, independent of established prognostic markers, though prognostic impact of low energy intake was preserved after adjustment for presence of cachexia.

The relationship between energy intake and the mortality is highly complex due to the cross-study heterogeneity including differences in study subjects (healthy vs. ill), study periods, and proportions of specific macronutrients (carbohydrates, protein, and fat), which cannot be easily adjusted for meta-analyses. In general, greater energy intake is associated with increased risk of mortality including risk of cardiovascular and cancer death in the general population and in patients with chronic diseases such as type 2 diabetes mellitus and end-stage CKD [17, 19, 30], whereas it is associated with lower mortality in critically ill patients with low BMI [31]. On the other hand, lower energy intake increases the risk of mortality in patients with chronic diseases [17, 19] and in elderly people [32], and a favorable effect of lower energy intake on longevity that has been observed in animal studies remains controversial in human studies [33, 34]. Thus, optimal energy intake for favorable prognosis seems to vary depending on patient characteristics.

The 2017 Academy of Nutritional and Dietetics (AND) published evidence-based nutritional practice guidelines in which the optimal amount of energy intake for improvement in quality of life is indicated [35]. However, there are no guidelines and statements showing appropriate daily energy intake for better prognosis in elderly HF patients since a systemic review of articles for the AND guidelines had been performed focusing on adults aged on 19 years or older with reduced LVEF (LVEF < 45%). On the other hand, guidelines for nutritional intervention in general older people were published by the ESPEN [36]. Total energy expenditure calculated by multiplying resting energy expenditure (20 kcal/kg) by physical activity factor (1.2–1.8) is 24–36 kcal/kg/day in older people. For this reason, approximately 30 kcal/kg/day of energy intake is recommended for older people and more than 30 kcal/kg/day may be appropriate for older people who are underweight for the purpose of fulfillment of energy requirement [36]. In the present study, the results of fully adjusted analysis showed a J-shaped relationship between energy intake and all-cause mortality, and risk of all-cause mortality was low among HF patients who consumed 25.0–40.0 kcal/kg /day of energy, supporting the ESPEN recommendation. As far as we know, the present study is the first study to suggest that there is optimal level of energy intake for improving prognosis in elderly HF patients. This issue clearly needs prospective study, though a favorable effect of nutritional support during hospital stay on clinical outcome including survival was reported [37].

HF patients with Low EI showed a tendency for larger body weight, resulting in high BMI. This association was also found in analyses in which energy intake per standard body weight and that per target body weight were used, being consistent with the results of a previous study in patients with type 2 diabetes mellitus [19]. This is an unexpected finding since HF patients with a higher BMI had a lower mortality rate than that in HF patients with normal or lower BMI, a phenomenon that has been termed the “obesity paradox” [38, 39]. There are several potential mechanisms to explain the unexpected association between low energy intake and high BMI. First, patients with Low EI had a greater severity of HF and a higher prevalence of CKD, contributing to congestion-induced increase in body weight and poor prognosis, though BNP levels and NYHA functional class, established markers of HF severity, were similar in the low, mid, and high EI groups. Second, patients with low EI might have high fat mass together with low muscle mass, i.e., sarcopenic obesity [8]. Importantly, sarcopenic obesity has been shown to be a predictor of all-cause mortality among elderly people, especially hospitalized patients [40]. Although the mechanisms underlying the development of sarcopenia obesity remain to be elucidated, chronic inflammation such as that induced by TNF-α and IL-6 signaling is a possible contributor to the development of sarcopenic obesity [41]. Detailed body composition analysis by the use of dual-energy X-ray absorptiometry together with cytokine measurements are needed to confirm this hypothesis. Nevertheless, assessment of energy intake has an impact on the prediction of mortality independent of BMI since the association between low energy intake and increased mortality remained after adjustment for BMI (Model 1).

There are limitations in the present study. First, since this study was a retrospective observational study using a small number of patients in a single center, there might have been selection bias in study subjects. In addition, differences in the effects of nutritional status and energy intake on the mortality between HF patients with different etiologies of heart failure were not analyzed because of insufficient statistical power. Second, the study subjects were patients who were admitted to our institute for diagnosis and/or treatment of chronic HF, and ambulatory patients were not included in the present study. Since proportions of HF patients with malnutrition were different in inpatients and ambulatory patients, it might not be possible to directly extrapolate findings in the present study to ambulatory HF patients [5, 6]. Third, HF patients who died in hospital could not be included in the analyses since nutritional status and daily energy intake were assessed during 3 consecutive days before discharge. Fourth, presence of cachexia was diagnosed by BMI reduction according to the criteria by Fearon et al. [21] in the present study, but presence of other factors associated with cachexic conditions, e.g., chronic inflammation and decrease in muscle strength and mass, may play a crucial role in prediction of survival, which are involved in the diagnostic criteria for cachexia by Evans et al. [42]. Thus, further analysis is needed to demonstrate the role of cachexia in prognostic impact of low energy intake in HF patients. Fifth, an obvious limitation in the present study is the lack of the analysis for the relationship between intake of macronutrients (carbohydrates, protein, and fat) and the mortality, though protein intake was positively correlated with energy intake in the present study (data not shown). Finally, there are race-dependent variations in anthropometric parameters including BMI. Thus, the results of the present study may not necessarily be applicable to other ethnicities.

## Conclusions

Energy intake during hospital stay predicts the all-cause death after discharge in elderly HF patients independent of established prognostic markers including nutritional status. Assessment of energy intake may be useful for further risk stratification of HF patients with malnutrition.
